# Impact of fracture morphology on the biomechanical stability of osteosynthetic fixation

**DOI:** 10.1007/s00068-025-02802-0

**Published:** 2025-03-20

**Authors:** Marianne Hollensteiner, Mischa Mühling, Philipp Blum, Sabrina Sandriesser, Dirk Baumeister, Markus Greinwald, Julian Fürmetz, Peter Augat

**Affiliations:** 1 Institute for Biomechanics, BG Unfallklinik Murnau, Prof. Küntscher Str. 8, 82418 Murnau, Germany; 2 Institute for Biomechanics, Paracelsus Medical University Salzburg, Strubergasse 21, Salzburg, 5020 Austria; 3 Department of Trauma Surgery, BG Unfallklinik Murnau, Prof. Küntscher Str. 8, 82418 Murnau, Germany; 4 Department of Orthopaedics and Trauma Surgery, Musculoskeletal University Center Munich (MUM), University Hospital, LMU Munich, Marchioninistraße 15, 81377 Munich, Germany; 5 Institute for Biomechanics, BG Unfallklinik Murnau and Paracelsus Medical University Salzburg, Prof. Küntscher Str. 8, 82418 Murnau, BG Germany

**Keywords:** Femur, Biomechanical assessment, Fracture pattern, Osteotomy

## Abstract

Biomechanical testing is essential for evaluating osteosyntheses, particularly in assessing stability, stiffness, and fragment motion. However, traditional flat-fracture models created via osteotomy fail to replicate the complex morphology of real-world fractures, potentially reducing the applicability of results. This study introduces patient-specific distal femur fracture models to investigate the impact of fracture morphology on the biomechanical performance of osteosyntheses. Realistic fracture models were generated using 3D printing and molding, based on CT-derived geometry, alongside traditional osteotomy models. Four groups were tested: osteotomized and realistic fracture models, with and without gaps. All constructs were treated with distal femur locking plates and subjected to axial and torsional loading. Dynamic testing simulated physiological conditions and tracked interfragmentary motions with a 3D optical motion system. Realistic fracture models exhibited higher torsional stiffness and reduced interfragmentary motion compared to osteotomized models, particularly in closed fracture gaps. Axial stiffness increased significantly upon fracture gap closure in all gap groups, transitioning from exclusively plate-bearing to construct-bearing configurations. The irregular geometry of realistic fractures provided enhanced interlocking, improving stability under both axial and torsional loads. Patient-specific fracture models better replicate the mechanical behaviour of clinical distal femur fractures, demonstrating advantages over osteotomized fracture models. The inclusion of realistic fracture geometries in biomechanical testing improves the transfer of biomechanical results into a clinical setting and offers valuable insights for optimizing designs and improving clinical outcomes.

## Introduction

Biomechanical testing is a cornerstone in evaluating and advancing osteosynthetic fixation methods. These tests are essential for assessing key parameters such as stiffness and fragment motion, which help predict potential complications including follow-up fractures, implant failure, or delayed healing and non-union [1, 2]. Traditionally, biomechanical evaluations utilize bone surrogates, either human specimens or synthetic bones, with standardized, flat-fracture surfaces created through osteotomy. While these models offer reproducibility and consistency, they often fail to accurately represent real fractures’ complex geometries and variability [3, 4]. This limitation reduces the clinical applicability of findings, as the simplified, plane fracture geometries in current models fail to replicate the interlocking properties and irregular stress distributions of real fractures [5, 6]. They simplify the fracture morphology, potentially overlooking the irregularities and complexities found in real-world fractures. Real fractures, including those in the distal femur, can exhibit a range of characteristics such as varying degrees of comminution, jagged or serrated fragments, or angular misalignment. These factors significantly influence the biomechanical performance of osteosynthetic devices and may lead to discrepancies between laboratory results and clinical outcomes [6, 7].

The distal femur, in particular, presents unique challenges due to its complex biomechanics and high load-bearing demands. Biomechanical testing is crucial for understanding the mechanical behavior of osteosyntheses in these challenging fractures, as implant failure and non-union remain significant complications. Implant failure can result from inadequate stability or excessive interfragmentary motion, while non-union often stems from suboptimal mechanical conditions, such as insufficient load sharing or stress shielding. Addressing these issues through improved biomechanical insights is critical for advancing surgical outcomes and reducing complication rates [8]. Fractures in this region are prone to complications such as malalignment, delayed union, and implant failure, making accurate biomechanical testing crucial for effective treatment planning [9–13]. Despite advancements in surgical techniques, such as angular stable plates and retrograde nails, complications continue to challenge outcomes due to the intricate nature of distal femur fractures [7, 14].

Addressing these challenges, a novel biomechanical realistic fracture model has been developed. These surrogates have undergone extensive validation to confirm their mechanical [15, 16] and morphological [17, 18] accuracy. Recent advancements have further enhanced these models to include realistic fracture patterns. The ability to individually mould and manufacture the surrogates allows for the incorporation of fracture geometries, derived from patient-specific CT scans or other clinically relevant fracture configurations. This approach facilitates the replication of a broader spectrum of fracture types and configurations commonly observed in clinical practice, with a specific emphasis on the distal femur in this study.

The objective of this investigation is to evaluate the biomechanical performance of osteosyntheses using realistic fracture models in comparison to osteotomized fracture models, with a specific focus on fractures of the distal femur. Key parameters assessed include axial and torsional stiffness as well as fragment motion.

## Materials and methods

### Patient-specific fractured distal femora

For this study, distal PuReBone femur models were used. The base materials of these synthetic bones are composed of polyurethane and various additives. The mixtures for both cortical bone [15] and cancellous bone [18] as well as the whole femora were already mechanically validated against human femora [16].

**Fig. 1:**
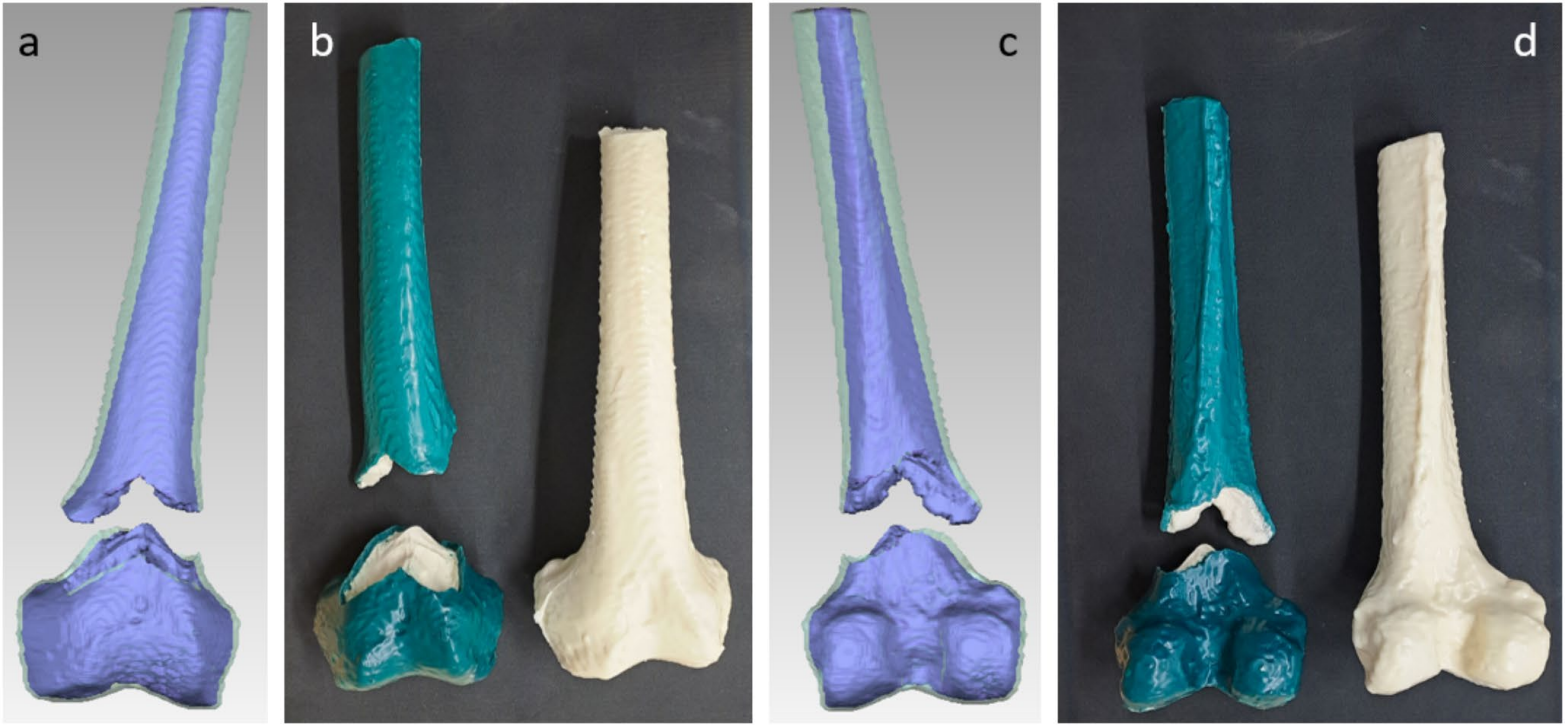
Views of the patient-specific fracture and the entire distal femur model (**a**: Anterior view of the digital model of patient-specific distal femur fracture. Blue proportion indicates cancellous bone, greenish proportion shows cortical encasing; **b**: Anterior view of patient-specific fracture model (left) and intact femur (right); **c**: Posterior view of digital model of patient-specific distal femur fracture. Blue proportion indicates cancellous bone, greenish proportion shows cortical encasing; **d**: Posterior view of patient-specific fracture model (left) and intact femur (right)

A real distal femur fracture (AO/OTA 33 A3) was segmented from a CT scan of a male patient using D2P Professional v1.03 (3D Systems GmbH, Mörfelden-Walldorf, Germany). Two femur models were created: one representing the fractured femur derived from the patient’s CT data, and another representing an intact femur. The intact femur was reconstructed by digitally reducing the fracture model (Geomagic Wrap v.2021.2.2, 3D Systems Inc., Rock Hill, USA), assembling the fragments, and smoothing the transitions. The cortical and cancellous components were segmented separately and 3D printed individually using a Raise3D N2 Plus printer (Raise 3D Technologies Inc., Irvine, CA, USA). These prints were used as negatives to create silicone moulds, which were subsequently employed to produce intact and fractured femora, consisting of synthetic cancellous bone encased in synthetic cortical bone (Fig. 1). The detailed manufacturing process of these femora is documented elsewhere [16].

### Fracture treatment

**Fig. 2:**
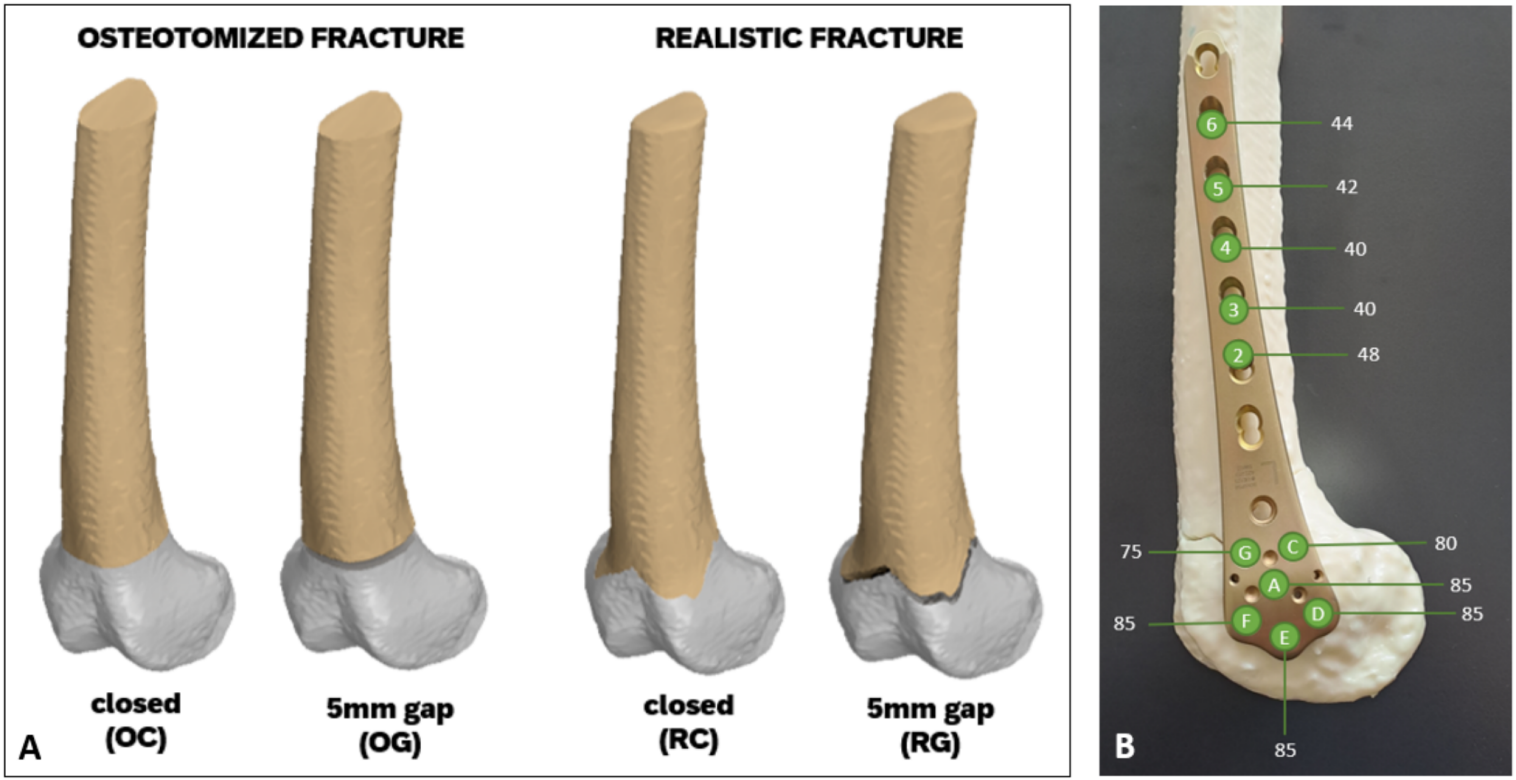
**A**: Overview of the four femur groups: OC (osteotomized femur without gap), OG (osteotomized femur with 5 mm gap), RC (realistic fractured femur without gap), and RG (realistic fractured femur with 5 mm gap). **B**: Screw placement configuration in the distal femur locking compression plate (LCP DF). The screw lengths used are indicated for each screw position

The patient-specific femurs were divided into four groups, with *n* = 8 samples each: osteotomized femur with a closed gap (OC), osteotomized femur with a 5 mm gap (OG), realistic fractured femur with a closed gap, (RC), and realistic fractured femur with a 5 mm gap (RG, Fig. 2A). All specimens were treated with 7-hole distal femur locking compression plates (LCP DF, Depuy Synthes Inc., Paoli, PA, USA) by an experienced surgeon. The anatomically pre-contoured plates were fitted with self-tapping locking screws (diameter 5 mm, varying lengths, titanium, Depuy Synthes, Paoli, PA, USA) in holes A, C, D, E, F, and G. Additionally, the shaft area was secured with self-tapping locking screws in holes 2 to 6 (Fig. 2B).

The fracture line was consistently positioned 6 cm proximal to the intercondylar notch [19, 20] matching the location of the real fracture. The AO/OTA 33 A3 fracture type was chosen due to its clinical relevance as an unstable distal femur fracture [21]. The 5 mm gap simulates non-anatomical reduction, a common scenario in complex fractures or delayed surgical treatment [22]. For the osteotomized fractures, this procedure was replicated using an oscillating saw (GOP 18 V-28, Robert Bosch GmbH, Gerlingen, Germany). Additionally, a cutting template ensured that all osteotomies were performed uniformly and reproducibly across the specimens. This process ensured that the osteotomized fracture models corresponded closely to the height of the realistic fracture line, maintaining consistency between the two approaches.

### Mechanical testing

#### Preparation of the samples

**Fig. 3:**
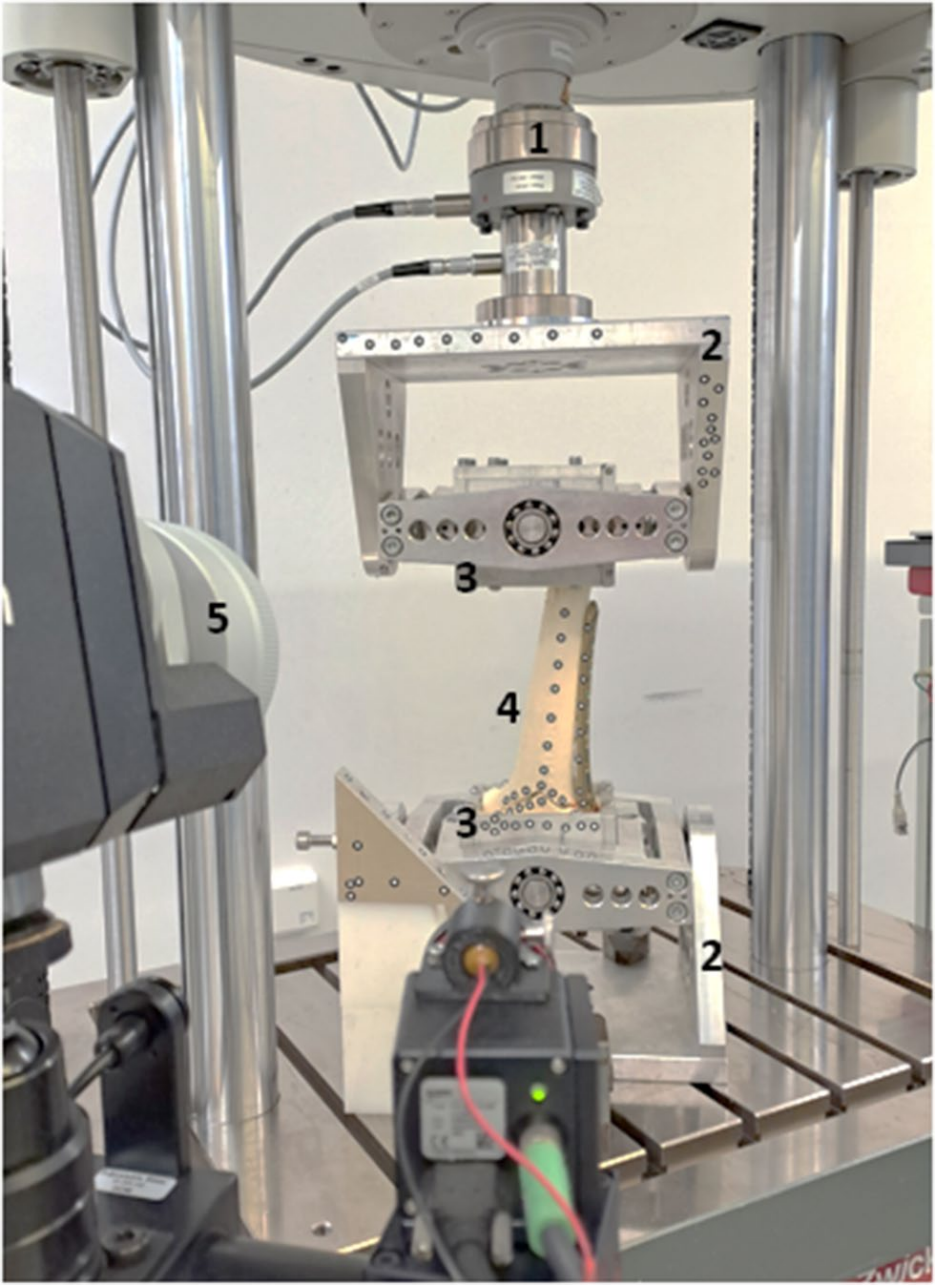
Test setup (1. force/torque actuator with load cell, 2. cardan joints, 3. embedding pots, 4. bone surrogate with adhesive markers, 5. 3D camera system)

After fracture fixation, the distal and proximal bone segments were embedded in fast-curing resin (GP010 casting resin, Gößl & Pfaff GmbH, Karlskron, Germany). To ensure free motion of the implants during testing, screws, and plates were covered with modelling clay (Noris, Staedlter Mars GmbH & Co. Kg, Nürnberg, Germany). The distal femur was embedded in a physiological 6° valgus position [23, 24] and secured distally and proximally into cardan joints [25] to prevent constrained forces and moments while testing in the testing machine (LTM Z010, ZwickRoell GmbH & Co. KG, Ulm, Germany, Fig. 3).

#### Measurement setups

The loading protocol comprises both static and dynamic testing phases to thoroughly evaluate the mechanical performance of the bone-implant constructs. First, the bone-implant constructs were subjected to axial loading at a ramp rate of 0.2 mm/s until 1500 N was reached [25]. The axial construct stiffness was determined by analyzing the slope of the load-deformation curve. For groups with a fracture gap (OG and RG), stiffness was calculated both before and after gap closure, while for groups with a closed fracture gap (OC and RC), stiffness was assessed only on a single occasion. Second, quasi-static torsional testing was also conducted using a ramped approach at a rotational speed of 0.15°/s until 4 Nm [25]. The torsional stiffness of the bone-implant construct was then calculated as the slope of the angle-torque curve, for positive and negative torsion, respectively, where positive torsion corresponds to internal rotation and negative torsion to external rotation of the femur.

The dynamic testing of bone-implant constructs involved a loading protocol to simulate realistic physiological conditions during gait. Torsional sinusoidal loading was applied at a frequency of 0.5 Hz, with alternating torques of ± 4 Nm, which mimic the external and internal rotational moments encountered in the knee during human gait in full weight bearing [26, 27]. Concurrently, axial loading was applied in a sinusoidal pattern at a frequency of 1 Hz, between 50 N (valley load) and 1500 N (peak load), replicating the dynamic compressive forces experienced during a patient’s full weight bearing. A total of 110 cycles were examined, with the initial 10 cycles used to gradually ramp up the loads and to settle the construct, followed by 100 measurement cycles [28].

After the cyclic test, a combined quasi-static axial/ torsional ramp was performed to assess fragment motion under maximum loading conditions. The ramps were executed such that the maximum axial load of 1500 N was simultaneously applied with either + 4 Nm or -4 Nm torsional load. This protocol allowed for precise measurement of fragment motions and deformation under peak combined loading scenarios, providing additional insights into the mechanical behaviour and stability of the bone-implant constructs.

#### Tracking interfragmentary motions with optical 3D motion system

Before mechanical testing, small self-adhesive point markers were applied along the fracture line, both on the proximal and distal sides of the fracture and along the shaft axis, the plate, and the test setup itself. To determine interfragmentary motions, these marker points were tracked by an optical 3D motion system (ARAMIS Professional 6 M, Zeiss GOM metrology GmbH, Braunschweig, Germany) in the unloaded and loaded states, respectively. The displacements and rotations of the fragments were calculated based on a coordinate system oriented in the frontal plane. The coordinate system was defined with the z-axis oriented along the actuator axis of the testing machine, pointing caudally. The y-axis represents the sagittal axis, pointing anteriorly. The x-axis is perpendicular to the y- and z-axes, representing the transverse axis, pointing laterally. The origin of the coordinate system was consistently positioned for all specimens to ensure reproducibility and comparability.

The relative motions of fracture fragments were assessed in three-dimensional space, with displacements and rotations evaluated along the z-axis—corresponding to the actuator load axis of the testing machine—and shear motion analyzed in the horizontal x-y plane. Measurements were taken at the fragment’s most medial point, representing the location of maximum tipping and fracture closure. This point, furthest from the osteosynthesis, is most sensitive to fragment motion and thus provides a robust stability evaluation.

### Data analysis

The statistical analysis was performed using SPSS (SPSS Statistics v.26, IBM, Amonk, USA). For stiffness, the pure axial and torsional ramps conducted before the cyclic test were evaluated. For interfragmentary motions, the combined ramps after the cyclic test were analyzed. Given that negative rotation in this test setup corresponds to external rotation of the femur in the knee joint, the results focus on this direction due to its biomechanical relevance: external rotation in the knee allows for greater rotational freedom (approximately 30°) compared to the restricted internal rotation (approximately 10°), which is limited by the tension in the cruciate ligaments. This greater range of motion during external rotation results in a higher torque compared to internal rotation [29]. Negative rotation was therefore considered, as this corresponds to the external rotation of the femur.

Kolmogorov Smirnov and Levene test were used to test for normal distribution and homogeneity of variances. Even though the tests confirmed a normal distribution, homoscedasticity could not be confirmed (*p* < 0.010). Thus, construct stiffnesses and fracture motions were compared using Welch-ANOVA, followed by Games-Howell post-hoc tests. Level of significance was set to 0.05.

## Results

### Axial construct stiffness

**Fig. 4:**
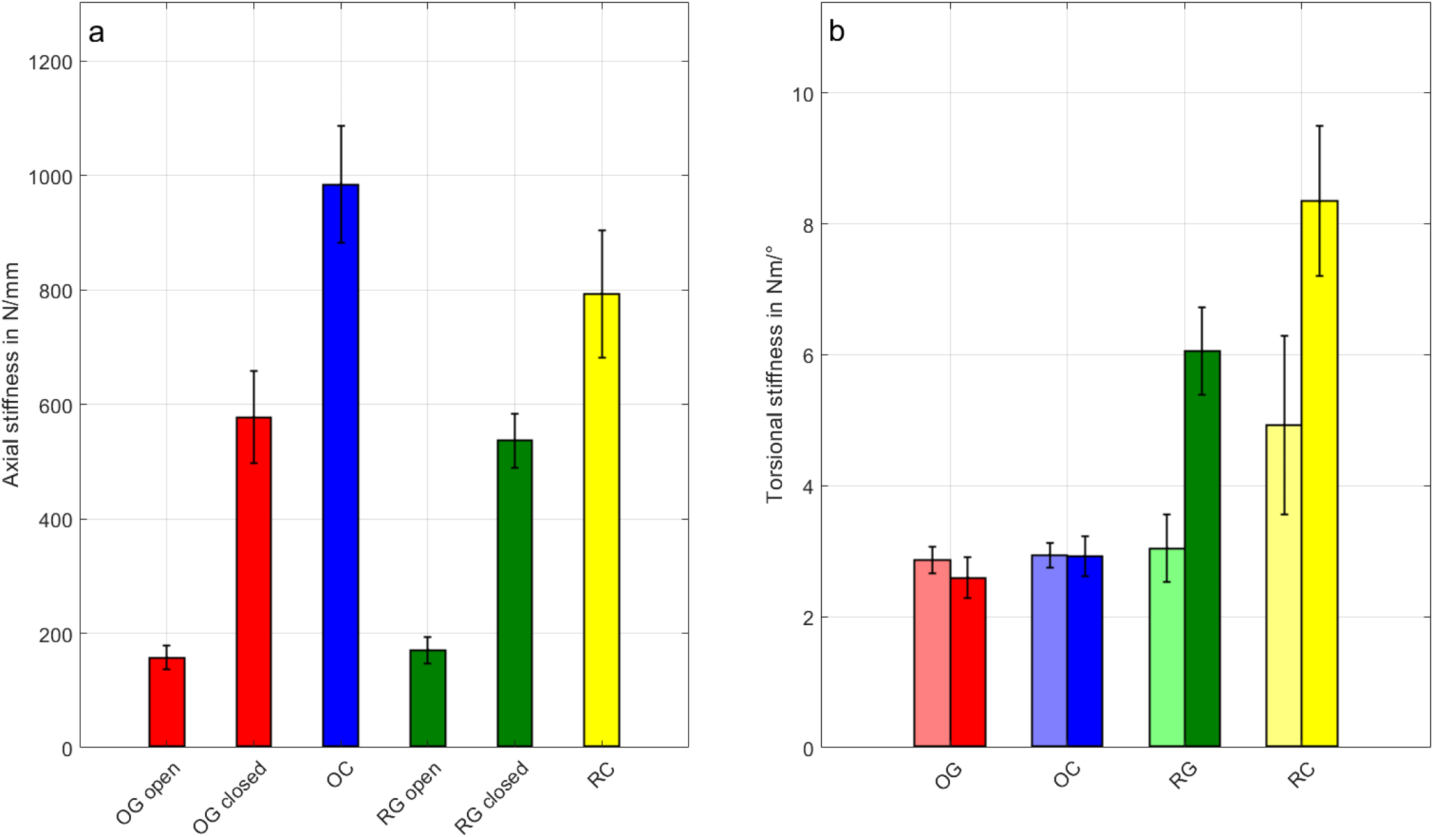
(**a**) Axial stiffness values. (**b**) Torsional stiffness values. Each bar represents the mean value, and the error bars indicate the standard deviation. Group colours are consistent between subplots, with OG (red), OC (blue), RG (green), and RC (yellow). Light color in the torsion plot indicates internal rotation while dark color represents external rotation

The axial stiffness of the fracture constructs varied distinctly based on the type of fracture configuration and the presence of a gap (Fig. 4a). Constructs with a gap (OG and RG) demonstrated a pronounced increase in stiffness upon closure of the fracture (*p* < 0.001), highlighting the mechanical contribution of gap closure under axial loading at around 1000 N. Specifically, OG constructs showed a 267% increase in stiffness when transitioning from the open (OG open) to the closed state (OG closed), reflecting the shift from an exclusively plate-bearing to a construct-bearing configuration. Similarly, RG constructs exhibited a 215% increase in stiffness from the open fracture state (RG open) to the closed state (RG closed). Statistical analysis confirmed significant differences in axial stiffness between the open and closed states of gap constructs (OG and RG, *p* < 0.001).

In the open state, OG constructs displayed slightly lower stiffness than RG constructs, with a difference of approximately 8%, likely due to variations in fracture treatment techniques by the surgeon rather than the mechanical influence of the irregular fracture edges in RG. Closed constructs without a gap (OC and RC) exhibited consistently higher axial stiffness, as the absence of a gap eliminated the transition phase associated with fracture closure. The OC configuration, characterized by smooth osteotomy edges, demonstrated an axial stiffness approximately 24% higher than RC constructs (*p* < 0.001), which featured real fracture edges that allowed for minor displacements.

### Torsional construct stiffness

The torsional stiffness of the constructs also varied significantly depending on the fracture configuration and the direction of rotation (Fig. 4b). Constructs with an osteotomy gap (OG) exhibited the lowest torsional stiffness across all groups, with no notable differences between positive and negative rotations (*p* = 0.563). Similarly, OC constructs, which featured smooth osteotomy edges without a gap, demonstrated comparable torsional stiffness to OG constructs under both positive and negative moments, indicating that the absence of interlocking mechanisms limits torsional stability.

In contrast, constructs with realistic fracture edges (RG and RC) exhibited pronounced directional asymmetry in torsional stiffness. The RG configuration demonstrated a substantial increase in stiffness for positive rotation compared to negative rotation, with a relative increase of approximately 99% (*p* < 0.001). Similarly, RC constructs showed a significant asymmetry, with stiffness under positive rotation approximately 70% higher than under negative rotation (*p* < 0.001). Statistical analysis revealed that RC constructs exhibited the highest torsional stiffness under positive rotation, significantly exceeding all other groups (*p* < 0.001). A similar pattern was observed in RG constructs, which displayed significantly higher stiffness under positive than negative rotation (*p* < 0.001). Additionally, RC and RG constructs demonstrated significantly greater stiffness under negative moments compared to OC and OG constructs (*p* < 0.001).

### Interfragmentary motions

**Fig. 5:**
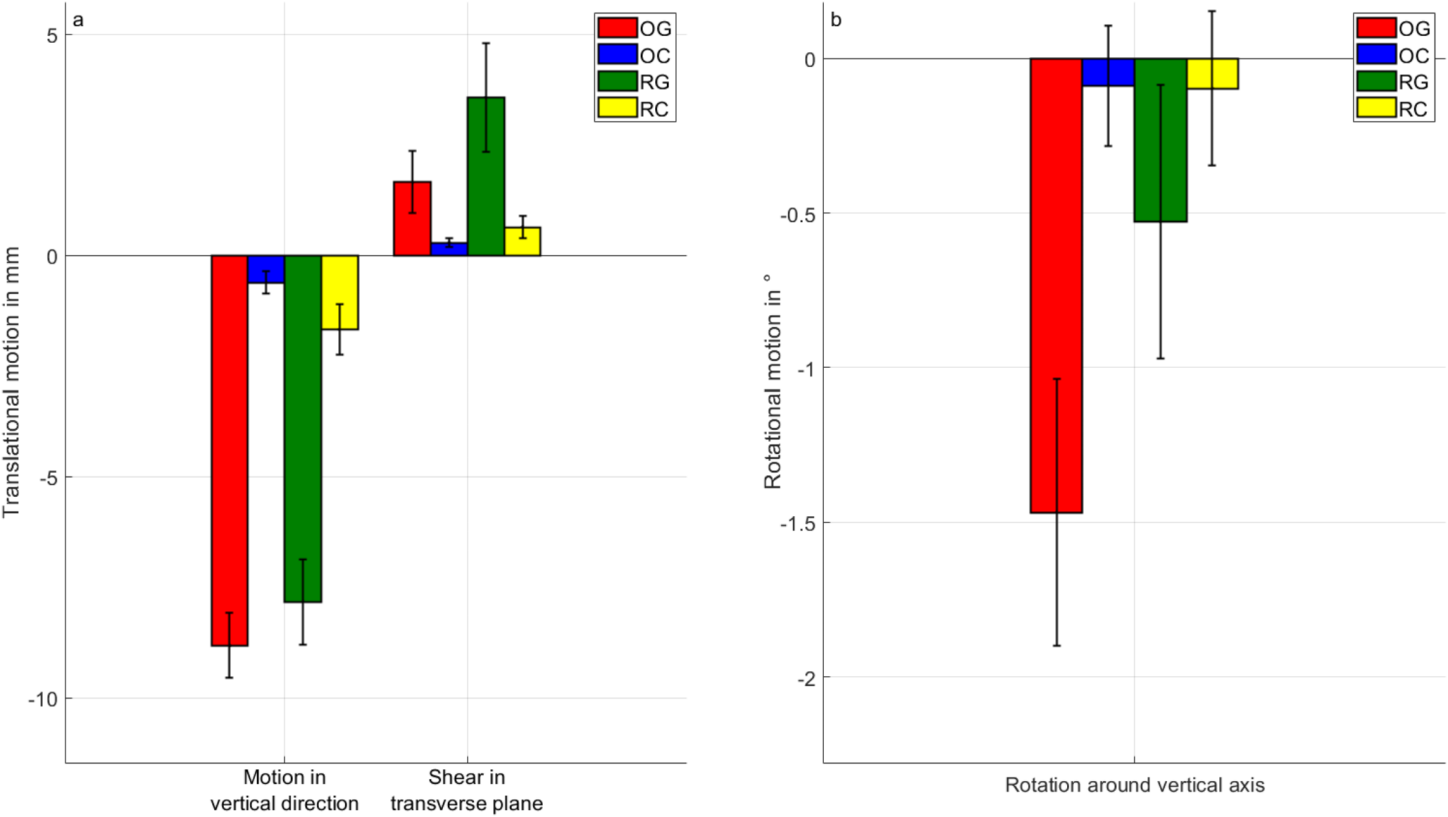
(**a**) Translational motion (in actuator-direction and shear in the transverse plane) in [mm]. (**b**) Rotational motion (around the vertical axis) in [ °]. The bars are colour-coded to indicate groups: OG (red), OC (blue), RG (green), and RC (yellow). Error bars indicate standard deviations

The analysis of interfragmentary motion revealed significant differences between the groups, reflecting the impact of fracture design and gap conditions on mechanical stability (Fig. 5). Translational displacements along the actuator’s direction were markedly reduced in the closed fracture groups (OC and RC) compared to their respective gap fracture counterparts (OG and RG) (*p* < 0.001). Specifically, OC exhibited a reduction in vertical motion of approximately 93% relative to OG, while RC demonstrated an 80% decrease compared to RG. Similarly, shear motion in the transverse plane was significantly diminished in the closed fractures, with OC showing an 83% reduction compared to OG and RC demonstrating an 82% decrease relative to RG.

Rotational motion around the shaft axis was also significantly reduced in the closed fracture groups. OC exhibited a reduction of approximately 94% in rotational motion compared to OG, while RC showed an 81% decrease relative to RG. Statistical analysis indicated significant differences in rotational motion around the loading direction of the actuator for most group comparisons (*p* ≤ 0.005), except for OC and RG, where differences were not statistically significant (*p* = 0.108). The reduced motion observed in closed fractures emphasizes the stabilizing effect of fracture closure, which was consistent across both osteotomized and realistic fracture designs.

## Discussion

The objective of this study was to evaluate the biomechanical performance of osteosyntheses by comparing realistic fracture models to traditional flat-fracture models, with a specific focus on the distal femur. The results revealed significant differences in biomechanical performance between the two fracture models. Constructs with realistic, interlocked fractures exhibited significantly higher torsional stiffness after fracture gap closure compared to flat, osteotomized fractures. Axial stiffness also differed significantly depending on the presence of gaps, while interfragmentary motions, particularly torsional motion, were significantly higher in flat fractures than in realistic ones.

This study highlights how variations in fracture morphology impact the biomechanical performance of osteosynthetic constructs. Realistic, interlocked fracture models consistently outperformed simplified, flat osteotomies in terms of mechanical stability, primarily due to their complex geometries and natural interlocking, which enhance load distribution and interfragmentary engagement. In contrast, flat osteotomies, lacking interlocking mechanisms, exhibited lower torsional stiffness and greater interfragmentary motion. Their oversimplified geometry fails to replicate the nuanced mechanical behaviour of clinical fractures, potentially compromising the validity of biomechanical tests.

The use of osteotomies in biomechanical studies is a widely accepted method for replicating fractures in a controlled and standardized manner. This approach facilitates precise replication of specific fracture scenarios, enabling systematic comparisons of osteosynthesis techniques and implants while minimizing variability and ensuring consistent results [3].

However, osteotomies lack the complexity of real fractures, which often include comminution, fragmentation, and irregular fracture lines. These features significantly influence fracture stability and are challenging to replicate using osteotomies. Consequently, the biomechanical behaviour of osteotomized fractures may differ substantially from real fractures, which exhibit more pronounced stress concentrations and irregular loading patterns due to their natural formation. A study on metacarpal bones, for instance, demonstrated that realistic fracture models more accurately simulate in vivo conditions, revealing differences in load and stiffness between monocortical and bicortical fixations that were not as pronounced in osteotomized models [30].

Although osteotomies can be tailored to replicate specific fracture types, they often fail to reproduce the in vivo loading conditions and dynamic environments of real fractures. For example, even advanced setups using proximal first metatarsal osteotomies in complete human specimens fall short of fully capturing the complexity of real fracture behaviour [31]. The cleaner, uniform cuts of osteotomies may perform better with certain fixation techniques, but these results may not translate well to fractures with more intricate geometries [32].

Schader et al. demonstrated that realistic pertrochanteric fracture models, created through controlled force application in human specimens in a Guillotine-like setup, outperform osteotomized models in biomechanical testing, offering greater homogeneity and enhanced stability under cyclic loading. The superior interlocking properties of the rough trabecular surfaces in realistic fractures improve their predictive value and clinical relevance. However, their study focused exclusively on stable pertrochanteric fractures, limiting their applicability to other fracture types. Furthermore, the artificially created fractures, while more representative than osteotomies, do not fully replicate the natural variability and pathomechanics of clinical fractures. Despite these limitations, their work highlights the potential of realistic fracture models to enhance the reliability and clinical applicability of biomechanical testing [6]. Additionally, real fractures also exhibit significant variability due to differences in bone quality, fracture location, and the presence of soft tissue damage. Factors such as age, sex, and ethnicity can further influence the anatomy and mechanical properties of bones, as demonstrated in previous research on femoral variability across diverse populations [33].

Axial stiffness varied with fracture morphology and gap conditions. Gap closure significantly increased stiffness, transitioning constructs from plate-bearing to construct-bearing load distributions. This suggests that while fracture gap and closure conditions strongly influence axial motion, the osteotomized and realistic gap fractures demonstrated comparable behaviour in this regard. While osteotomized gap models showed slightly higher stiffness in the open state, this advantage disappeared upon gap closure. Closed constructs consistently demonstrated superior stiffness, with smoother osteotomy edges providing enhanced stability compared to realistic fractures with irregular surfaces.

Torsional behaviour further underscored the importance of fracture morphology. Realistic fractures exhibited directional asymmetry in torsional stiffness, reflecting their interlocking resistance to rotation features absent in flat osteotomies. This uniform torsional behaviour suggests that the presence of a gap minimises the effects of fracture morphology on torsional stability and leads to comparable stiffness in both directions of rotation, as only the stiffness of the plate contributes to the construct stiffness.

Realistic gap constructs outperformed osteotomized gap models in resisting rotational forces, emphasizing the biomechanical relevance of interlocking mechanisms. Interfragmentary motion analysis confirmed the advantages of realistic fracture models, which exhibited reduced translational and rotational motion compared to osteotomized models. Reduced motion is crucial for maintaining alignment and creating favorable conditions for healing [34]. Closed fracture configurations demonstrated higher stability compared to open fractures with a gap, as shown in this study and supported by previous findings [35]. However, the lack of statistical differences in certain rotational metrics suggests a complex interplay between fracture morphology and construct mechanics.

The findings underscore the enhanced stiffness and reduced motion of realistic fracture models, highlighting their ability to emulate clinical fracture mechanics more effectively than simplified osteotomies. These interlocking characteristics improve load resistance and mechanical stability, supporting their use in biomechanical testing. Standardized testing conditions using realistic fracture models improve reproducibility and reliability, ensuring that implants are evaluated under conditions that closely mimic clinical scenarios, ultimately enhancing their design and performance [6].

While this study introduces a novel biomechanical model, several limitations must be addressed. The use of synthetic bone models, though mechanically and morphologically validated, may not fully replicate the properties of human bone. While the synthetic bone models used in this study are highly customizable and can replicate parameters such as anatomy, sex, population-specific traits, bone density, and osteoporosis status [16, 36], they still represent only a snapshot of a single population. This limitation restricts the ability to fully capture the variability seen in human bone across different demographics and conditions. Additionally, the absence of biological factors such as vascularization, soft tissue interactions, and dynamic healing responses may simplify the mechanical environment, potentially impacting the generalizability of the findings.

However, these synthetic bones have been rigorously validated in prior studies, demonstrating their suitability as surrogates for human femora in biomechanical testing [15–18]. This ensures that the mechanical insights drawn from this study are robust and reliable.

A critical limitation of biomechanical testing, including the present study, is the exclusion of muscles and fasciae, which play a significant role in fracture stability. Muscles and fasciae generate dynamic forces that influence the mechanical environment of osteosyntheses, potentially altering both stiffness and interfragmentary motion [37, 38]. While this aspect remains largely unexplored in biomechanical research, it represents an important area for future studies. The use of our custom-made synthetic bones provides the flexibility to incorporate simulated muscle forces by embedding or attaching structures that mimic muscular tension. Such integration is currently part of ongoing research efforts within our team, aiming to better replicate in vivo conditions and further enhance the clinical relevance of biomechanical findings.

The biomechanical tests, while replicating physiological loading conditions, may not capture the complex, multi-directional forces occurring in vivo; furthermore, fracture morphology can be even more complicated in vivo, as fractures are often multifragmentary. The experimental setup does not fully mimic daily activities, however, combined axial and torsional loads mimic a physiologic load scenario. Moreover, focusing on a single fracture type (AO/OTA 33 A3) in the distal femur, restricts the generalizability of results to other fracture types and anatomical locations. The behaviour of osteosynthetic constructs may vary significantly in fractures with different characteristics (e.g., comminuted or open fractures) or in bones subject to different loading environments (e.g., the tibia or humerus).

The observed differences between realistic and osteotomized fracture models emphasize the need for a nuanced approach to biomechanical testing. Realistic fractures, with higher stiffness and reduced torsional motion, may be less prone to mechanical failure, potentially reducing the risk of complications such as implant failure or non-union. Excessive torsional motion in osteotomized fractures may impair healing, highlighting the need to consider fracture morphology in device design [39]. This highlights the importance of considering fracture morphology in the selection and design of osteosynthetic devices, as devices optimized for flat osteotomized fractures may not perform as well in fractures with more complex geometries.

The inherent variability in biomechanical testing must also be acknowledged. The choice of testing models- synthetic bone surrogates, or human specimens- can significantly affect outcomes and their clinical relevance. While synthetic models allow controlled comparisons, they fail to replicate the biological and mechanical variability of in vivo conditions, challenging the validity of findings [40].

Expanding this research to include a broader range of fracture types and locations would provide a more comprehensive understanding of fracture morphology’s impact on biomechanical performance. Future clinical studies correlating these findings with patient outcomes will be essential for translating biomechanical insights into improved care.

## Conclusion

In conclusion, this study highlights fracture morphology’s critical role in determining osteosynthetic constructs’ biomechanical performance. The use of realistic, patient-specific fracture models provides a more accurate representation of the clinical scenarios encountered in orthopedic practice, offering valuable insights that could inform the design and optimization of osteosynthetic devices. While further research is needed to fully understand the implications of these findings, this study represents an important step toward improving the accuracy and relevance of biomechanical assessments in orthopedics and biomechanics. Additionally, given the increasing focus on enabling immediate full weight-bearing, particularly in geriatric patients, it is essential to consider that previous fracture models may have been overly conservative, underscoring the need for more representative and progressive approaches in future research.

## Data Availability

Research data will be made available on request.
